# Burnout Among Health Professionals Working in Intensive Care Units of Southern Ethiopia: A Multicenter Cross-Sectional Study

**DOI:** 10.3389/ijph.2025.1608337

**Published:** 2025-04-28

**Authors:** Seyoum Hailu, Mesfin Gurmu, Gose Husen, Adamu Tesfaye, Birhanu Muleta, Henok Yeshitila, Belayneh Bekele

**Affiliations:** ^1^ Department of Anesthesiology, Dilla University, Dilla, Ethiopia; ^2^ School of Medicine, Dilla University, Dilla, Ethiopia; ^3^ Department of Emergency and Critical Care Nursing, Dilla University, Dilla, Ethiopia

**Keywords:** burnout, ICU, intensive care unit, health professionals, Ethiopia

## Abstract

**Objective:**

This study aimed to investigate the prevalence and associated factors of burnout among health professionals working in intensive care units.

**Methods:**

After receiving ethical clearance from the institutional review board of Dilla University College of Health Sciences with protocol unique number *duirb/033/23-05*, a multicenter cross-sectional study was conducted. Binary and multivariate logistic regressions were used to assess the relationship between burnout syndrome as dependent and various personal job factors as independent factors.

**Results:**

The overall prevalence of BOS among HCPs working in the selected university hospitals of southern Ethiopia is 38.1%. Health professionals who worked at night duty were 4.15 times more likely to be in a state of burnout as compared to those who were on day duty shift [AOR = 4.15, 95%CI (1.27–13.58)].

**Conclusion:**

Burnout is a great public health concern. Age, marital status, workload, working the night shift, fear of patient death, less quipped setup, and absence of extra-time duty payment were among the predictive variables.

## Introduction

Burnout is a state of emotional exhaustion, depersonalization, and reduced sense of accomplishment that can affect people who work closely with others [1, 2]. Burnout arises from prolonged exposure to chronic emotional stressors at work, leading to mood disturbances, mental health issues, physical ailments, and a negative outlook on professional responsibilities. It can also result in poor communication with patients and various complications in both social and organizational aspects of life [3, 4].

Burnout is a concept that individuals may interpret in various ways. Before Maslach provided a standardized definition, people used the term to describe different experiences. In 1982, Maslach defined burnout as a condition marked by emotional exhaustion, depersonalization, and a diminished sense of personal accomplishment. This state commonly affects individuals who work closely with others, stemming from prolonged exposure to stress associated with human interaction [5, 6]. Healthcare professionals are on the front lines of patient care and report some of the highest rates of burnout syndrome, with roughly 40% of hospital nurses experiencing burnout levels that exceed those of other healthcare workers [3, 7]. Working in an intensive care unit is particularly stressful due to high rates of patient morbidity and mortality, demanding daily routines, and frequent encounters with critical situations [8–11]. Extended exposure to high levels of stress can lead to various health issues, with burnout being the most common and immediate concern. Its prevalence ranges from 20% to 60% among healthcare professionals [12].

Burnout increases clinicians’ risk of negative professional and personal outcomes, including reduced free time, a higher likelihood of work-home conflicts, increased depression, and lower career satisfaction [13]. Burnout is a work-related issue that impacts nearly every area of a healthcare professional’s life. It is linked to physical illness, emotional challenges, higher turnover rates, workplace absenteeism, reduced job performance, and negative attitudes. Together, these issues create a long list of difficulties that ultimately diminish the quality of patient care [14].

The intensive care unit (ICU) is a high-pressure environment within hospitals, providing specialized critical care to patients, which contributes to a heavy workload for healthcare professionals, a key factor in increasing the risk of burnout. The presence of advanced medical equipment, the severity of patients’ conditions, long working hours, and other stressors all contribute to heightened stress levels among staff. Maintaining the wellbeing of healthcare professionals is essential for ensuring quality patient care. A meta-analysis and systematic review showed that within the dimensions of burnout, emotional exhaustion had a significant relationship with depression and personality factors [15]. Another meta-analysis and systematic review which was done in Ethiopia revealed the overall proportion of Burn out syndrome among health professionals is 32.73%. Both sociodemographic factors and working conditions influence the risk of burnout syndrome [16].

While numerous studies on burnout have been conducted in Europe, the US, Australia, parts of Africa, and Asia, there is limited research on this topic, particularly in Ethiopia. Specifically, no study has examined the prevalence of burnout in intensive care units in Ethiopia. Therefore, this study aimed to assess the prevalence of burnout and identify the factors associated with it among healthcare professionals working in ICUs at randomly selected university hospitals in southern Ethiopia.

## Methods

A multicenter cross-sectional study was conducted to assess the prevalence and associated factors of burnout among health professionals working in intensive care units from 09/11/2023 to 09/05/2024GC in three selected teaching and university hospitals of southern Ethiopia, which includes Dilla University, Hawassa University, and Wolaita Sodo University Hospitals. While all health professionals working in the intensive care unit of the selected hospitals were considered as the study population, our sample population considers only health professionals who are available during the study period. All health professionals with work experience of 6 months and above, and were available during the data collection period and willing to participate in the study were included while those who were on sick, study, or other leaves during the data collection period, those who were on grief in the last 2 months of the data collection period, and those who were providing free services for the hospitals were considered to be in the exclusion criteria. This study was done in line with the World Medical Association Declaration of Helsinki’s Ethical Principles for Medical Research Involving Human Subjects [17]and the work has been reported in line with the STROCSS 2021 criteria [18]. The study was also registered on the research registry with the unique identifying number of researchregistry10827 (https://www.researchregistry.com/browse-the-registry#home/).

### Sample Size Determination and Sampling Procedure

The sample size was determined by using the single population proportion formula assuming the p = 14% from a previous study done on the prevalence of burnout in ICU healthcare professionals (HCP) [19] since there was no study done on health professionals in ICU setting in Ethiopia with a 5% margin of error (d) and 95% confidence interval of certainty (alpha = 0.05), non-response rate 10%. Where, ${\;n=\frac{{\left(\frac{Z\alpha }{2}\right)}^{2\;}p\left(1-p\right)}{d^{2}}}^{2}$ Where: n = minimum sample size required for the study, Z = standard normal distribution (Z = 1.96) with a confidence interval of 95% and ⍺ = 0.05, we took P = 14% (0.14) since the overall prevalence of burnout was 14% in the previous study. d = Absolute precision or tolerable margin of error (d) = 5% = 0.05, Non response = 10%

Applying the formula: Then, n = $\frac{\left(1.96^{2}\right)\left[\left({\left(1-0.14\right)}^{2}0.14\right)\right]}{\left(0.05\right)2}=185$

The calculated sample size is 185.

By adding a 10% non-response rate the final sample size was = **204**. The total numbers of healthcare professionals working in intensive care units in southern Ethiopia, specifically in the three hospitals were included in the study. Therefore 44 participants were selected from DURH, 83 from HUCSH, and 77 were from WSURH. Based on eligibility criteria a total of two hundred-four participants were recruited consecutively until the sample size was achieved from the selected study areas.

### Data Collection Procedures

The data was collected by six trained nurses using a standardized questionnaire adapted from previous literature. The questionnaire was divided into three parts. The first section included 17 items that covered the sociodemographic and job-related details, the second portion contains lists of 19 symptoms of burnout syndrome (BOS) which are obtained from the Astudillo and Mendinueta and are rated from 0 (never), 1 (sometimes), 2 (often), and 3 (always). The sum of the scores of the rating of the items was calculated with a total minimum score of 0 to a maximum score of 57. Healthcare professionals who scored more than 23 were considered burned out [20]. The third portion of the questionnaire contained the Maslach Burnout Inventory (MBI) [21, 22] which was the most commonly used 22-item measuring tool to self-assess the risk of burnout and it is a validated tool that values the three syndrome subscales of emotional exhaustion, depersonalization, and lack of personal accomplishment. The items were scored on a Likert scale from 0 (never) to 7 (everyday) for which the participants were not aware of the scoring system and the BOS assessment scores were calculated as a summation of answers to each item and represented with their number and frequency. The questionnaires were given to the participants in all three study areas to fill during their free time after explaining the contents, aim, and anonymity of the study. There was no identifier for any personal response and the participants were not aware of the scoring system.

### Data Quality Assurance

All questions were administered in English, and the questionnaire was tested for reliability, psychometric properties, and internal validity. The internal consistency reliability was assessed using Cronbach’s alpha score for each section, with scores above 0.75, indicating strong construct reliability. Additionally, a pretest was conducted on 5% of the sample size, with adjustments made to suit the local context. The pretest results were excluded from the main study. During data collection, regular supervision and follow-up were maintained to ensure quality.

### Data Processing and Statistical Analysis

Data was checked, coded, and entered into Epi-info version 7.0 and transported to SPSS version 25 for analysis. Descriptive statistics was used to summarize tables and figures and numeric data was described in terms of number and frequency. Shapiro Wilk tests was employed for the normality test. Binary and multivariate logistic regression was used to assess the relationship between BOS as dependent and various personal job factors as independent factors. All Variables showing significance on bivariate analysis at a p-value less than 0.25 were taken to multivariate analysis one after the other to investigate model prediction and independent predictors of the explanatory variables. In multivariate analysis, a p-value of less than 0.05 was considered for the statistical association.

### Ethical Considerations

Ethical clearance and approval was obtained from the institutional review board of Dilla University College of Health Science with a protocol unique number of *duirb/033/23-05*. A support letter was obtained from the medical director’s office of each hospital for conducting this project. Informed consent was obtained from each participant during the eligibility assessment. All the information was kept confidential, and no individual identifiers were collected.

## Results

### Socio-Demographic Characteristics

Initially, a total of 204 participants were planned to be included in this study while two participants withdrew from the study and this makes a 99% response rate. Out of the whole respondents, the majority of them were male 139 (68.8%), and in the age range of 25–30. Nearly half the respondents were married and had 1–5 children while protestant was the highest religion.

The majority of the respondents were ICU nurses 56 (27.7%), while ethnic Oromos were higher in number. Regarding the respondent’s overall working year of experience, most of them 72 (35.6%) had 1–5 years of experience (Table 1).

**TABLE 1:** Socio-demographic characteristics (Southern Ethiopia, 2024).

Variables	Category	Frequency	Percent
Age	18–24	29	14.4
	25–30	74	36.6
	31–35	28	13.9
	36–45	52	25.7
	>46	19	9.4
Sex	Male	139	68.8
	Female	63	31.2
Marital status	Single	87	43.1
	Married	96	47.5
	Divorced	19	9.4
Number of children they have	None	74	36.6
	1–5	98	48.5
	More than 5	30	14.9
Religion	Orthodox	78	38.6
	Protestant	85	42.1
	Muslim	25	12.4
	Catholic	8	4.0
	Others	6	3.0
Ethnicity	Gedeo	37	18.3
	Sidama	24	11.9
	Wolaita	55	27.2
	Oromo	48	23.8
	Amhara	28	13.9
	Others	10	5.0
Type of Health Profession	Medical intern	22	10.9
	GP	25	12.4
	Anesthetist	26	12.9
	ICU nurse	56	27.7
	Residents	24	11.9
	Internists	24	11.9
	Others	25	12.4
Year of experience	Less than 2 years	48	23.8
	2–5 years	72	35.6
	More than 5	82	40.6

### Organizational and Work-Related Variables

Among all respondents majority of them treated less than 5 patients per day while more than half of the respondents were working at night duty. Most of them 127 (62.9%) did not work at private health institutions as a per-timer, there was work overload in more than half of them and most of the respondents 120 (59.4) had a fear of patient death while working in the ICU.

Most of the respondents 150 (74.3) complained that they didn't pay for extra time duty on time while around 126 (62.4%) of them explained that the working environment was not equipped with adequate medical equipment/Lab investigations/Medications. The majority of the respondents explain that their organization supports the staff by involving them in decision-making/decentralizing decision-making (Table 2).

**TABLE 2:** Work-related and organizational-related factors (Southern Ethiopia, 2024).

Variable	Category	Frequency	Percent
Number of ICU patients treated per day	<5 Pts	91	45.0
	5–10 pts	86	42.6
	>10 pts	25	12.4
What is your current duty shift?	Day shift	98	48.5
	Night shift	104	51.5
Do you work in private health institutions?	Yes	75	37.1
	No	127	62.9
Presence of work overload	Yes	109	54.0
	No	93	46.0
Do you have a fear of patient death while working in the ICU?	Yes	120	59.4
	No	82	40.6
Are you paid for any extra load/duty payment on time?	Yes	52	25.7
	No	150	74.3
Is your working environment equipped with adequate medical equipment/Lab investigations/Medications?	Yes	76	37.6
	No	126	62.4
How does the organization support the staff regarding your service?	Regular training	51	25.2
	Collaborative team meetings	26	12.9
	Providing an opportunity for advancement	25	12.4
	Giving positive feedback or rewards	10	5.0
	Involving the staff in decision-making/decentralizing decision-making	56	27.7
	Develop clear and accurate job descriptions	34	16.8

### Prevalence of Burnout Syndrome

The overall prevalence of BOS among HCPs working in the selected university hospitals of southern Ethiopia is 38.1% (scored more than 23 points on the Astudillo and Mendinueta 19-item scale). The majority of the participants stated that they have always experienced professional stress symptoms such as irritability (48.5%), debility (44.1%), and self-criticism (37.6%) while symptoms such as insomnia (40.6%), Fatigue (45%), depressive states (41.1%), social isolation (42.1%), problem with rest of the team (34.7%) and dissatisfaction with work (39.1%) were observed sometimes in majority of the respondents. Symptoms such as consuming analgesics (69.8%) and consuming sedatives (65.8%) were not detected in most of the respondents (Table 3).

**TABLE 3:** Burnout symptoms among healthcare professionals working in intensive care units of selected university hospitals (Southern Ethiopia, 2024).

S.No	Symptoms	Response			
		Never N (%)	Often N (%)	Sometimes N (%)	Always N (%)
1	Irritability	11 (5.4)	38 (18.8)	55 (27.2)	98 (48.5)
2	Debility	31 (15.3)	40 (19.8)	42 (20.8)	89 (44.1)
3	Self-criticism	39 (19.3)	45 (22.3)	42 (20.8)	76 (37.6)
4	Insomnia	49 (24.3)	58 (28.7)	82 (40.6)	13 (6.4)
5	Fatigue	15 (7.4)	52 (25.7)	91 (45.0)	44 (21.8)
6	Spinal problem	79 (39.1)	42 (20.8)	30 (14.9)	51 (25.2)
7	Lack of organization	19 (9.4)	116 (57.4)	43 (21.3)	24 (11.9)
8	Lack of sense of priority	77 (38.1)	73 (36.1)	26 (12.9)	26 (12.9)
9	Depressive states	34 (16.8)	50 (24.8)	83 (41.1)	35 (17.3)
10	Feeling of failure	35 (17.3)	94 (46.5)	35 (17.3)	38 (18.8)
11	Painful symptoms	64 (31.7)	78 (38.6)	43 (21.3)	17 (8.4)
12	Social isolation	33 (16.3)	49 (24.3)	85 (42.1)	35 (17.3)
13	Poor concentration and performance	64 (31.7)	89 (44.1)	22 (10.9)	27 (13.4)
14	Less caring attitude	23 (11.4)	99 (49.0)	40 (19.8)	40 (19.8)
15	Problem with rest of team	50 (24.8)	43 (21.3)	70 (34.7)	39 (19.3)
16	Dissatisfaction with work	42 (20.8)	28 (13.9)	79 (39.1)	53 (26.2)
17	Change of profession	40 (19.8)	92 (45.5)	48 (23.8)	22 (10.9)
18	Consume analgesics	141 (69.8)	46 (22.8)	8 (4.0)	7 (3.5)
19	Consume sedative	133 (65.8)	51 (25.2)	14 (6.9)	4 (2.0)

When comparing MBI subscales among HCPs, from the overall study participants high level of emotional exhaustion 82 (40.6%) and depersonalization 59 (29.2%) was observed while half of the respondents scored a low level of personal achievement 102 (50.5%) (Table 4).

**TABLE 4:** Maslach burnout inventory (Southern Ethiopia, 2024).

Sub-scales	Low	Moderate	High
EE	18 (8.9%)	102 (50.5%)	82 (40.6%)
DP	45 (22.3%)	98 (48.5%)	59 (29.2%)
PA	102 (50.5%)	26 (12.9%)	74 (36.6%)

### Factors Associated With Burnout

The relationship between independent variables and burnout was examined using both bivariate and multivariable analyses. Variables that demonstrated an association with the outcome variables at a p-value of ≤0.25 in the bivariate analysis were identified as candidates for inclusion in the multivariable logistic regression model. The multivariable logistic regression analysis accounted for all candidate variables simultaneously, and only the following variables were found to be significantly and independently associated with burnout.

Respondents aged 36–45 years, marital status (specifically married and divorced healthcare professionals), night shift duty, and delayed payment demonstrated a statistically significant positive association with the outcome variable. Conversely, the absence of workload and a lack of fear regarding patient mortality in the ICU exhibited a significant negative association with burnout syndrome.

Those aged 36–45 years were 13.20 times more likely to experience burnout compared to those in the younger age group of 18–24 years [AOR = 13.20, 95% CI (1.88–92.86)]. Married and divorced individuals were more likely to develop burnout compared to single individuals (p < 0.05). Health professionals working night shifts were 4.15 times more likely to experience burnout compared to those on day shifts [AOR = 4.15, 95% CI (1.27–13.58)]. Additionally, the absence of timely payment for overtime duties increased the risk of burnout by 8.29 times [AOR = 8.29, 95% CI (1.78–38.59)].

Participants who did not fear patient death in the ICU were 0.05 times less likely to develop burnout compared to those who reported fearing patient death [AOR = 0.05, 95% CI (0.01–0.23)]. Similarly, healthcare professionals who reported an absence of workload were 0.29 times less likely to experience burnout compared to those facing work overload [AOR = 0.29, 95% CI (0.08–1.03)]. However, variables such as the number of children and years of professional experience were not identified as significant predictors of burnout in the multivariate logistic regression analysis (Table 5).

**TABLE 5:** Multivariate analysis of predictor variables for burnout in selected university hospitals (Southern Ethiopia, 2024).

Variables	Category	Burn out		COR (95%CI)	AOR (95%CI)	P-value
		Yes	No			
Age	18–24	8	21	1	1	
	25–30	17	57	0.78 (0.29–2.08)	2.59 (0.38–17.68)	0.332
	31–35	12	16	1.97 (0.65–5.95)	1.25 (0.14–11.59)	0.845
	36–45	28	24	3.06 (1.15–8.16)	13.20 (1.88–92.86)	**0.010**
	>46	12	7	4.50 (1.31–15.52)	8.94 (0.95–84.50)	0.056
Marital status	Single	12	75	1	1	
	Married	54	42	8.04 (3.87–16.69)	91.72 (6.68–1,259.31)	**0.001**
	Divorced	11	8	8.59 (2.87–25.71)	55.95 (2.91–1,074.28)	**0.008**
Number of children	None	18	56	1	1	
	1–5	44	54	2.54 (1.31–4.92)	0.16 (0.01–2.07)	0.162
	More than 5	15	15	3.11 (1.28–7.59)	0.90 (0.13–6.38)	0.916
Years of experience	Less than 2 years	11	37	1	1	
	2–5years	23	49	1.58 (0.69–3.64)	2.74 (0.52–14.34)	0.233
	More than 5	43	39	3.71 (1.67–8.26)	3.7 (0.44–31.26)	0.230
Current Shift	Day	19	79	1	1	
	Night	58	46	5.24 (2.78–9.87)	4.15 (1.27–13.58)	**0.019**
Fear of patient death in ICU	Yes	56	64	1	1	
	No	21	61	0.39 (0.21–0.73)	0.05 (0.01–0.23)	**<0.0001**
Payment on time	Yes	6	46	1	1	
	No	71	79	6.89 (2.78–17.10)	8.29 (1.78–38.59)	**0.007**
Presence of workload	Yes	56	53	1	1	
	No	21	72	0.28 (0.15–0.51)	0.29 (0.08–1.03)	0.055
Adequately equipped setup	Yes	16	60	1	1	
	No	61	65	3.52 (1.83–6.76)	35.70 (3.93–323.83)	**0.001**

ICU-Intensive care Unit, COR-Crude Odds ratio, AOR-Adjusted Odds Ratio. Bold values indicates that statistically significant on multivariable analysis.

## Discussion

In this study, we aimed to determine the prevalence of burnout among healthcare providers in three university hospitals located in the southern region of the country. While numerous studies on burnout have been conducted worldwide, including several in Ethiopia, most of the Ethiopian studies have focused on the central or northern regions. Our research specifically targeted burnout in intensive care units due to the limited literature available in this area. Our findings showed that 38.1% of healthcare professionals exhibited symptoms of burnout syndrome (BOS). This prevalence is notably higher than the 13.7% reported in a study conducted in the northwestern region of Ethiopia [23] and another study done in southwestern Ethiopia which reported a prevalence of 34% [7] but lower than another study conducted in Ethiopia among Nurses which found the prevalence of BOS to be around 42.5% [24]. The prevalence of BOS in this current study is also higher than the findings in studies done in Iran [25] and Brazil [3] which showed a BOS prevalence of 21.9% and 10.1% respectively. The discrepancy among these findings may be due to focusing on a specific group of HCPs, the timing, and the difference in the setup of the healthcare system.

Our recent findings revealed professional stress symptoms, including irritability, fatigue, self-criticism, insomnia, exhaustion, depressive states, social isolation, difficulties with team dynamics, and job dissatisfaction. However, the use of sedatives and analgesics was uncommon among the participants. These results align with those of a similar study conducted in Qatar [26] on primary healthcare physicians which explains that Fatigue (94.6%) was the most common symptom, irritability (81.1%), dissatisfaction with work (69.6%), self-criticism (78%), depressive symptoms (77.9%) and insomnia (77.9%) while considering changing profession and using sedatives were uncommon (14.9% and 16.6% respectively). Unlike our findings in this Qatar study, it was common to use analgesics (89.3%) by physicians. This variability in analgesic usage might be due to local differences in how to handle painful conditions and differences in cultural perspective.

There were different scales used in different studies to determine the prevalence of burnout syndrome. Though we used a list of symptoms (19 items) obtained from Astudillo and Mendinueta [27] to determine the level of BOS, we also compared MBI subscales among HCPs. From the overall study participants, a high level of emotional exhaustion 82 (40.6%) and depersonalization 59 (29.2%) were observed while half of the respondents scored a low level of personal achievement 102 (50.5%). This finding was lower than another study done in Ethiopia [28] and higher than a study done in Spain [29] which showed the estimated prevalence of high levels of emotional exhaustion to be 21%, high levels of depersonalization at 30%, and low levels of personal achievement 44%. Another study in China on ICU nurses also found a relatively low MBI subscale score by detecting high-degree burnout (43.2%) in the emotional exhaustion subscale, followed by 41.2% in the personal accomplishment subscale and 26.1% in the depersonalization subscale [30]. The probable justification for this difference may be due to the difference in study setting, study population and tools, and methodological differences. In our study, we collected data at three University hospitals that are busy and this exposed the health professions to be in a high state of burnout. On the other hand majority of health professionals from developed countries like Spain might have good educational status, job titles, and strong social support, whereas, in our study, there were respondents with low social support, fatigue syndrome, psychological distress, and major sleeping problem which could probably expose for the development of burnout syndrome.

Several significant predictors were identified in this study. Respondents aged 36–45 were found to be 13.2 times more likely to experience burnout syndrome (BOS) compared to those under 30 years old. This may be attributed to increased social and economic challenges as age advances, combined with the demands of a high-pressure work environment. Younger professionals were perceived as more energetic and less susceptible to BOS. However, this finding contrasts with the results of a study conducted in China [30] which showed younger groups were more exposed to burnout. In a study done in Brazil [31] also older age was associated with lower emotional exhaustion and depersonalization scores and with higher professional accomplishment scores. The difference in setup, economic status, and social factors may contribute to this difference.

Other predictors identified in this study included marital status and night shift work. Being married or divorced emerged as a strong predictors of burnout, while working night shifts was found to increase the risk of burnout by 4.15 times. Night shifts disrupt the circadian rhythm, compromise rest and sleep, and disturb physiological balance. These findings are consistent with those of a study conducted in Iran [25]. Fear of patient death and delayed payments were also significant predictors of burnout while having a well-equipped working environment showed an inverse relationship with burnout. Individuals who experienced fear of patient death were more prone to stress and burnout. Additionally, workload was identified as a key factor contributing to burnout, often linked to an imbalance between the healthcare provider-to-patient ratio and the planned standards—a persistent challenge in many developing countries, including Ethiopia. These findings are consistent with results from other studies [7, 32–34]. Health facilities should be adequately equipped, as insufficient resources can lead to frustration among healthcare professionals. Furthermore, with the current global inflation and its particularly severe impact in Ethiopia, delays in timely payment for overtime duties emerged as a strong predictor of burnout. Our finding contrasts with the results of a study conducted in France [35] as we did not find gender and category of profession as predictive variables. Since burnout is an ongoing process that is associated with demographic characteristics and other environmental factors caution should be taken when comparing the results originating from different studies.

### Strengths

Our recent study had several strengths, including its multicenter design and its distinction as the first of its kind in Ethiopia to focus on healthcare professionals working in ICUs. Additionally, we addressed new variables not explored in previous studies, such as workplace setup and payment-related issues for healthcare professionals.

### Limitations

As a cross-sectional study, it could not establish cause-and-effect relationships between variables. Moreover, the use of self-administered questionnaires, even with validated tools, might have introduced overestimation or underestimation, leading to potential bias.

### Conclusion

This study revealed that a significant proportion of healthcare professionals (38.1%) experienced burnout, highlighting it as a major public health concern. Predictive factors included age, marital status, workload, night shifts, fear of patient death, inadequately equipped facilities, and lack of timely overtime payments. Given that the wellbeing of ICU healthcare professionals is crucial to the quality of care provided to critically ill patients, investigating burnout levels in this population is essential to draw attention to their challenges.

### Recommendation

We recommend that policymakers and administrators address these contributing factors and work towards creating a more supportive and conducive working environment.

### Challenges Encountered

The primary challenge encountered during the data collection process was the difficulty of translating certain medical terms in the questionnaire into the local languages. This issue was successfully addressed through consultations with language experts.

## Data Availability

Data is provided within the article or supplementary material.

## Abbreviations

BOS, Burnout syndrome; ICU, Intensive Care Unit; HCP, Healthcare Professionals; PACU, Post-Anesthesia Care Unit; DURH, Dilla University Referral Hospital; HUCSH, Hawassa University Comprehensive Specialized Hospital; WSUCSH, Wolaita Sodo University Comprehensive Specialized Hospital.

## References

[1] Montero-Marín J García-Campayo J. A Newer and Broader Definition of Burnout: Validation of the “Burnout Clinical Subtype Questionnaire (BCSQ-36).”. BMC Public Health (2010) 10:1–9. 10.1186/1471-2458-10-302 20525178 PMC2887826

[2] Fajardo-Lazo FJ Mesa-Cano IC Ramírez-Coronel AA Quezada FCR. Professional Burnout Syndrome in Health Professionals. Arch Venez Farmacol y Ter (2021) 40(3):248–55. 10.5281/zenodo.5038655

[3] Ribeiro VF Filho CF Valenti VE Ferreira M De Abreu LC De Carvalho TD Prevalence of Burnout Syndrome in Clinical Nurses at a Hospital of Excellence. Int Arch Med (2014) 7(1):22–7. 10.1186/1755-7682-7-22 24860618 PMC4031323

[4] Selič P Ignjatovič T Journal ZK-SM. 2012 Undefined. Burnout Among Slovenian Family Medicine Trainees: A Cross-Sectional Study. vestnik.szd.si (2022). Available online at: http://vestnik.szd.si/index.php/ZdravVest/article/view/567.

[5] Maslach C. Burnout: The Cost of Caring. (2003).

[6] Maslach C Jackson MPL SE, editors. Maslach Burnout Inventory Manual. 3rd ed. Consulting Psychologists Press (1996). Available online at: monkeypuzzletraining.co.uk/burnout-inventory-results.

[7] Belay AS Guangul MM Asmare WN Bogale SK Manaye GA. Prevalence and Associated Factors of Burnout Syndrome Among Nurses in Public Hospitals, Southwest Ethiopia. Ethiop J Health Sci (2021) 31(3):543–52. 10.4314/ejhs.v31i3.11 34483611 PMC8365496

[8] Mealer M. Burnout Syndrome in the Intensive Care Unit Future Directions for Research. Ann Am Thorac Soc (2016) 13(7):997–8. 10.1513/AnnalsATS.201604-280ED 27388395 PMC5802691

[9] Ageel M. Assessment of Occupational Burnout Among Intensive Care Unit Staff in Jazan. Saudi Arabia , Using the Maslach Burnout Inventory (2022). 10.1155/2022/1298887 PMC9034942 35469166

[10] Liyew B Tilahun AD Kassew T. Practices and Barriers towards Physical Assessment Among Nurses Working in Intensive Care Units: Multicenter Cross-Sectional Study. Biomed Res Int (2021) 2021:5524676. 10.1155/2021/5524676 34337020 PMC8294977

[11] Teixeira C Ribeiro O Fonseca AM Carvalho AS. Burnout in Intensive Care Units - a Consideration of the Possible Prevalence and Frequency of New Risk Factors: A Descriptive Correlational Multicentre Study. BMC Anesthesiol (2013) 13:38. 10.1186/1471-2253-13-38 24172172 PMC3826848

[12] Elbarazi I Loney T Yousef S Elias A. Prevalence of and Factors Associated with Burnout Among Health Care Professionals in Arab Countries: A Systematic Review. BMC Health Serv Res (2017) 17(1):491–10. 10.1186/s12913-017-2319-8 28716142 PMC5513024

[13] Brown S Liran A Visentin D Zilinsky I Kornhaber R. Burnout and Compassion Fatigue: Prevalence and Associations Among Israeli Burn Clinicians (2017). p. 1533–40. 10.2147/NDT.S133181 PMC5478274 28670122

[14] Loundou A Embriaco N Azoulay E Barrau K Kentish N Pochard F High Level of Burnout in Intensivists. Am J Respir Crit Care Med (2007) 175:686–92. 10.1164/rccm.200608-1184oc 17234905

[15] Ramírez-Elvira S Romero-Béjar JL Suleiman-Martos N Gómez-Urquiza JL Monsalve-Reyes C Cañadas-De la Fuente GA Prevalence, Risk Factors and Burnout Levels in Intensive Care Unit Nurses: A Systematic Review and Meta-Analysis. Int J Environ Res Public Health (2021) 18(21):11432. 10.3390/ijerph182111432 34769948 PMC8583312

[16] Mengist B Amha H Ayenew T Gedfew M Akalu TY Assemie MA Occupational Stress and Burnout Among Health Care Workers in Ethiopia: A Systematic Review and Meta-Analysis. Arch Rehabil Res Clin Transl (2021) 3(2):100125. 10.1016/j.arrct.2021.100125 34179761 PMC8212011

[17] Association WM. World Medical Association Declaration of Helsinki: Ethical Principles for Medical Research Involving Human Subjects. JAMA (2013) 310(20):2191–4. 10.1001/jama.2013.281053 24141714

[18] Mathew G Agha R for the SG Goel P Mukherjee I Pai P Strocss 2021: Strengthening the Reporting of Cohort, Cross-Sectional and Case-Control Studies in Surgery. Int J Surg (2021) 96:106165. 10.1016/j.ijsu.2021.106165 34774726

[19] Mera MJF Gaspar RV García IZ Sánchez SV. Síndrome de burnout en distintas Unidades de Cuidados Intensivos. Enfermería intensiva (2009) 20(4):131–40. 20038381 10.1016/s1130-2399(09)73221-3

[20] Astudillo WMC Mendinueta C. Exhaustion Syndrome in Palliative Care. Support Care Cancer (1996) 4:408–15. 10.1007/bf01880637 8961470

[21] Maslach C Jackson S Leiter M. Maslach Burnout Inventory (1997). Available online at: https://psycnet.apa.org/record/1997-09146-011.

[22] Score T. Burnout Self-Test Maslach Burnout Inventory (MBI) (2021). p. 1–3.

[23] Bhagavathula AS Gebreyohannes EA Gebresillassie BM Bm G. Burnout Syndrome Among Healthcare Professionals Working in Gondar University Hospital, Northwest Ethiopia: A Cross-Sectional Study. Value Health (2017) 20:A884. 10.1016/j.jval.2017.08.2634

[24] Zewdu T Abera H Abebe N Mulugeta H Dessie G. Level of Burnout and Associated Factors Among Nurses Working in Public Health Institutions North Shoa Zone, Amhara, Ethiopia (2017) 5(8):17–26. Available online at: www.ijournals.in.

[25] Mohammadpoorasl A Maleki A Sahebihagh MH. Prevalence of Professional Burnout and its Related Factors Among Nurses in Tabriz in 2010. Iran J Nurs Midwifery Res (2012) 17:524–9. 23922600 PMC3730457

[26] Al-kuwari MG Health P Corporation C. Prevalence and Determinants of Burnout Syndrome Among Primary Healthcare Physicians in Qatar. (2015).

[27] Amour LL. Exhaustion Syndrome in Palliative Care (1996). p. 408–15. 10.1007/BF01880637 8961470

[28] Adbaru DG Assen ZM Demelew TM Teshome GS. Magnitude of Burnout and its Associated Factors Among Nurses Working in Public Hospitals of Amhara Regional State. Ethiopia (2024).

[29] Vargas C Vargas C Can GR. Risk Factors and Prevalence of Burnout Syndrome. In: The Nursing Profession International Journal of Nursing Studies Risk Factors and Prevalence of Burnout Syndrome in the Nursing Profession (2015). 10.1016/j.ijnurstu.2014.07.001 25062805

[30] Zhang X Huang D Guan P. Job Burnout Among Critical Care Nurses from 14 Adult Intensive Care Units in Northeastern China: A Cross-Sectional. survey (2014). 10.1136/bmjopen-2014-004813 PMC4067832 24948747

[31] Carlotto MS. Burnout Syndrome and Associated Factors Among Health Professionals of a Public Hospital. (2011). 10.1590/s2237-60892012000200008 25922928

[32] Tekeletsadik S Mulat H Necho M Waja T. Occupational Stress and its Associated Factors Among Health Care Professionals Working at a Setting of a Specialized Mental Hospital, Addis Ababa, Ethiopia, 2017: A Hospital-Based Cross-Sectional Study. J Psychol Psychother (2020) 10(1):1–8. 10.35248/2161-0487.20.10.368

[33] Zewdu AT Abera H Abebe N Dessie G. Level of Burnout and Associated Factors Among Nurses Working in Public Health Institutions North Shoa Zone. Amhara (2017) 5(8):17–26.

[34] Girma B Nigussie J Molla A Mareg M. Health Professional’s Job Satisfaction and its Determinants in Ethiopia: A Systematic Review and Meta-Analysis. Arch Public Heal (2021) 79(1):141–11. 10.1186/s13690-021-00664-7 PMC8340440 34353375

[35] Moukarzel A Michelet P Durand A Sebbane M Bourgeois S Markarian T Burnout Syndrome Among Emergency Department Staff. Prevalence and Associated Factors (2019). 10.1155/2019/6462472 PMC6360614 30800675

